# Reconstruction of Active Regular Motion in Amoeba Extract: Dynamic Cooperation between Sol and Gel States

**DOI:** 10.1371/journal.pone.0070317

**Published:** 2013-08-05

**Authors:** Yukinori Nishigami, Masatoshi Ichikawa, Toshiya Kazama, Ryo Kobayashi, Teruo Shimmen, Kenichi Yoshikawa, Seiji Sonobe

**Affiliations:** 1 Department of Life Science, Graduate School of Life Science, University of Hyogo, Harima Science Park City, Hyogo, Japan; 2 Department of Physics, Graduate School of Science, Kyoto University, Kyoto, Japan; 3 Department of Mathematical and Life Sciences, Graduate School of Science, Hiroshima University, Kagamiyama, Higashi-Hiroshima, Japan; 4 Faculty of Life and Medical Sciences, Doshisha University, Kyotanabe, Kyoto, Japan; University of Cambridge, United Kingdom

## Abstract

Amoeboid locomotion is one of the typical modes of biological cell migration. Cytoplasmic sol–gel conversion of an actomyosin system is thought to play an important role in locomotion. However, the mechanisms underlying sol–gel conversion, including trigger, signal, and regulating factors, remain unclear. We developed a novel model system in which an actomyosin fraction moves like an amoeba in a cytoplasmic extract. Rheological study of this model system revealed that the actomyosin fraction exhibits shear banding: the sol–gel state of actomyosin can be regulated by shear rate or mechanical force. Furthermore, study of the living cell indicated that the shear-banding property also causes sol–gel conversion with the same order of magnitude as that of shear rate. Our results suggest that the inherent sol–gel transition property plays an essential role in the self-regulation of autonomous translational motion in amoeba.

## Introduction

**Figure 1:**
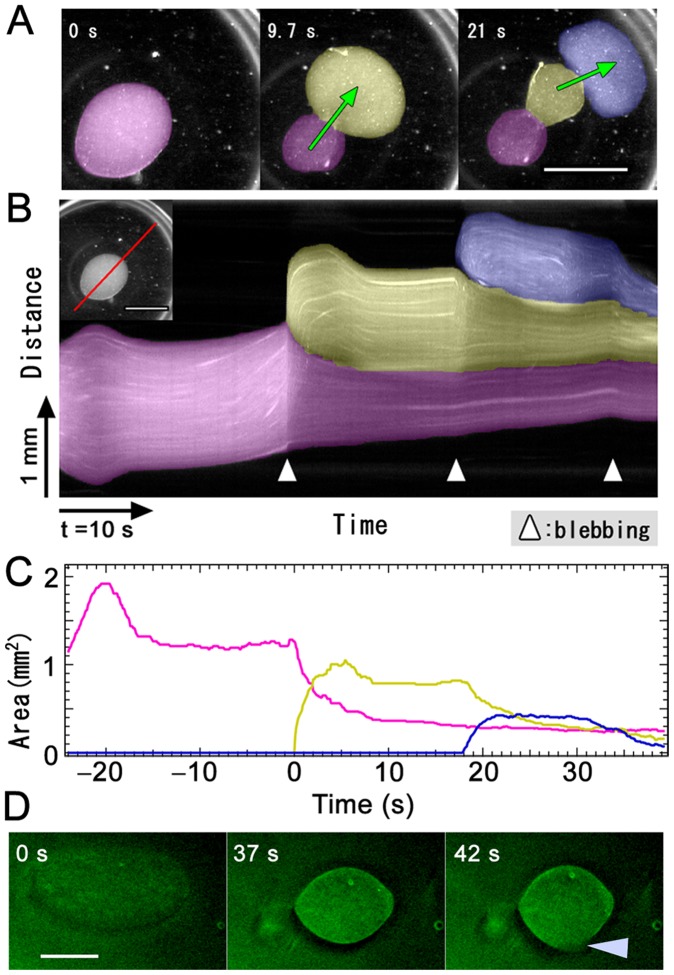
Observation of the IVA system. (A) On injecting the actomyosin fraction (pseudocolor) into cytosolic extract (clear region), the actomyosin fraction showed movement resembling bleb-driven amoeboid locomotion. Scale bar, 1 mm. (B) Kymograph of a moving IVA system. (C) Temporal changes in each pseudopod-like structure area. (D) Distribution of actin in IVA system. Just after injecting actomyosin fraction into cytoplasmic extract, actin was diffused in entire IVA system. After a few tens of seconds, actin accumulated at the boundary surface between cytoplasmic extract and actomyosin fraction. At the time of forming a new pseudopod like structure, a part of the boundary surface actin was broken (arrow head) and actomyosin body content effused into the surrounding cytosolic extract from the broken boundary surface. Scale bat, 500 µm.

Amoeboid locomotion, the most typical mode of locomotion in adherent eukaryotic cells, appears in biological processes such as embryonic development [1], wound healing [2], and cancer metastasis [3]. Actin polymerization in the elongating part generates a direct driving force in locomotion [4]. However, the amoeboid locomotion mechanism “bleb-driven amoeboid locomotion” has been proposed recently [5], [6], in which the cell cortex actomyosin contracts to increase hydrostatic pressure inside the cell and part of the cell cortex is broken in the desired direction of flow. This type has been recognized in many cell types during cell migration within a three-dimensional matrix or *in vivo* tissue [5]. During bleb-driven amoeboid locomotion, cytoplasmic sol and gel states must be spatiotemporally regulated. If the cortex strength and gel and sol configuration are uncontrolled, the cell cannot migrate in the desired direction. Because cytoplasm mainly comprises actin and the state of actin is regulated by actin-binding proteins in a calcium ion-dependent manner *in vitro*, calcium ions are considered important for cytoplasmic sol–gel conversion [7]. Furthermore, microscopic analyses have revealed that the amoeba cytoplasm has a sol layer enclosed by a gel layer [8] (Figure S1 and Movie S1). During locomotion, the sol layer flows in the direction of locomotion, with the gel layer fixed. The sol in the anterior region is then transferred to the gel in parallel with cytoplasmic motion cessation. Although sol–gel conversion phenomena have been studied since the early 20th century [8], its mechanistic basis remains unclear because of bleb-driven amoeboid locomotion complexity. Therefore, a novel model system was developed (Figure 1, S2, Movie S2 and S3) in which the essence of bleb-driven amoeboid locomotion was isolated from the cell complexity. Despite keen interest in bleb-driven amoeboid locomotion, motility has never been reconstituted *in vitro*. However, the reconstructed system accurately demonstrates phenomenological aspects of efficient *in vivo* motion regulation. Reconstitution approaches have been adopted to understand complex biological phenomena such as the cell cycle [9], mitotic spindle assembly [10], eukaryotic cilia and flagella beating [11], and filopodia elongation [12]. Here, the reconstitution of bleb-driven amoeboid locomotion using a crude cytosolic extract and an actomyosin fraction from *Amoeba proteus* is reported. Rheological studies in the model system and *in vivo* suggest that shear stress regulates the cytoplasmic sol and gel states. This inherent rheological property could regulate gel and sol cytoplasm formation during cell locomotion.

## Materials and Methods

### Cell Culture

We performed mass culture of *Amoeba proteus*, as described previously [13]. In brief, *Amoeba proteus* was cultured in KCM medium (7 mg/l KCl, 8 mg/l CaCl_2_, and 8 mg/l MgSO_4_ · 7H_2_O) at 25°C and fed with *Tetrahymena pyriformis*. Cells were starved for at least 2 days before use to avoid contamination with *T. pyriformis*.

### Preparation of *in vitro* Amoeba (IVA) System

We carried out all preparations at 2°C and observed movement at room temperature. Ten grams of *A. proteus* was suspended in an EMP solution (2 mM EGTA, 2 mM MgCl_2_, 20 mM PIPES–KOH, pH 7.0) and centrifuged at 6,000×g for 2 min and after removal of the supernatant at 600,000×g for 20 min to obtain a cytosolic extract. The precipitate was suspended in a 3 M KCl solution (3 M KCl, 2 mM MgCl2, 1 mM DTT, 20 µg/ml leupeptin, 20 µg/ml pepstatin A, 20 mM imidazole-HCl, pH 7.0) and centrifuged at 400,000×g for 10 min. The resultant supernatant was dialyzed against 50 mM KCl, 2 mM EGTA, 2 mM MgCl_2_, 1 mM DTT, 0.2 mM ATP, 20 mM imidazole-HCl, pH 7.0 for 5 h. Actomyosin was collected by centrifugation at 20,000×g for 5 min and suspended in 150 µl EMP buffer containing 1 mM DTT to prepare an actomyosin fraction. ATP at a final concentration of 2 mM was added to either the amoeba extract or the actomyosin fraction or both. Concentrations of proteins in both the fractions were determined by the method of Bradford, and the concentrations of both were approximately 20 mg/ml. One microliter of the actomyosin fraction was injected into 10 µl of the cytosolic extract on a cover glass. Movement was observed by microscopy (Olympus SZH) in a dark field equipped with a CCD camera (Victor KY-F550).

### Treatment with Inhibitors

To examine the effects of inhibitors, we added 5 µM latrunculin B, 30 µM cytochalasin D, and 100 mM 2,3-butanedione monoxime to both fractions on ice with 0.1% dimethyl sulfoxide (DMSO) and allowed to stand for 15 min before injection. A circularity ratio was calculated by length of boundary surface and area of actomyosin fraction using ImageJ and Igor 6.22A (WaveMetrics).

### Electron Microscopy

For electron microscopy, we injected 1 µl of the actomyosin fraction into 10 µl of the cytosolic extract in a test tube and carefully added 1 ml of a fixative solution (10% glutaraldehyde, 0.5% tannic acid, and 50 mM cacodylate buffer pH 7.0) to the samples. The samples were postfixed with 2% OsO4 in cacodylate buffer (pH 7.0) for 1 h, dehydrated, and embedded in Spurr’s resin. Ultrathin sections were stained with uranyl acetate and lead citrate and observed using an electron microscope (JOEL JEM 1200 EX).

### Fluorescence Time-laps Imaging

To reveal the distribution of actin in the IVA system, we stained cytosolic extract and actomyosin fraction by Alexa Flour 488 phalloidin (invitrogen). Time-laps Images were taken by Microscopy (Leica MZ 16F) with a CCD camera (Olympus DP72).

### Rheological Studies

We processed a thin silicon sheet (TIGARS POLYMER SR-200) into a chamber using a cutting machine (GRAPHTEC Craft ROBO pro). We used a real-time confocal system (Olympus IX71, Yokogawa CSU-X1 and Andor iXon+) to obtain a fluorescence image of the actomyosin fraction containing fluorescent microspheres (Invitrogen FluoSpheres polystyrene microspheres 1 µm) and *A. proteus* stained with MitoTracker Deep Red FM (Invitrogen). Data analyses were performed using ImageJ and Igor 6.22A (WaveMetrics).

### Statistical Analysis

Statistical analysis was carried out using Igor 6.22A (WaveMetrics).

## Results and Discussion

**Figure 2:**
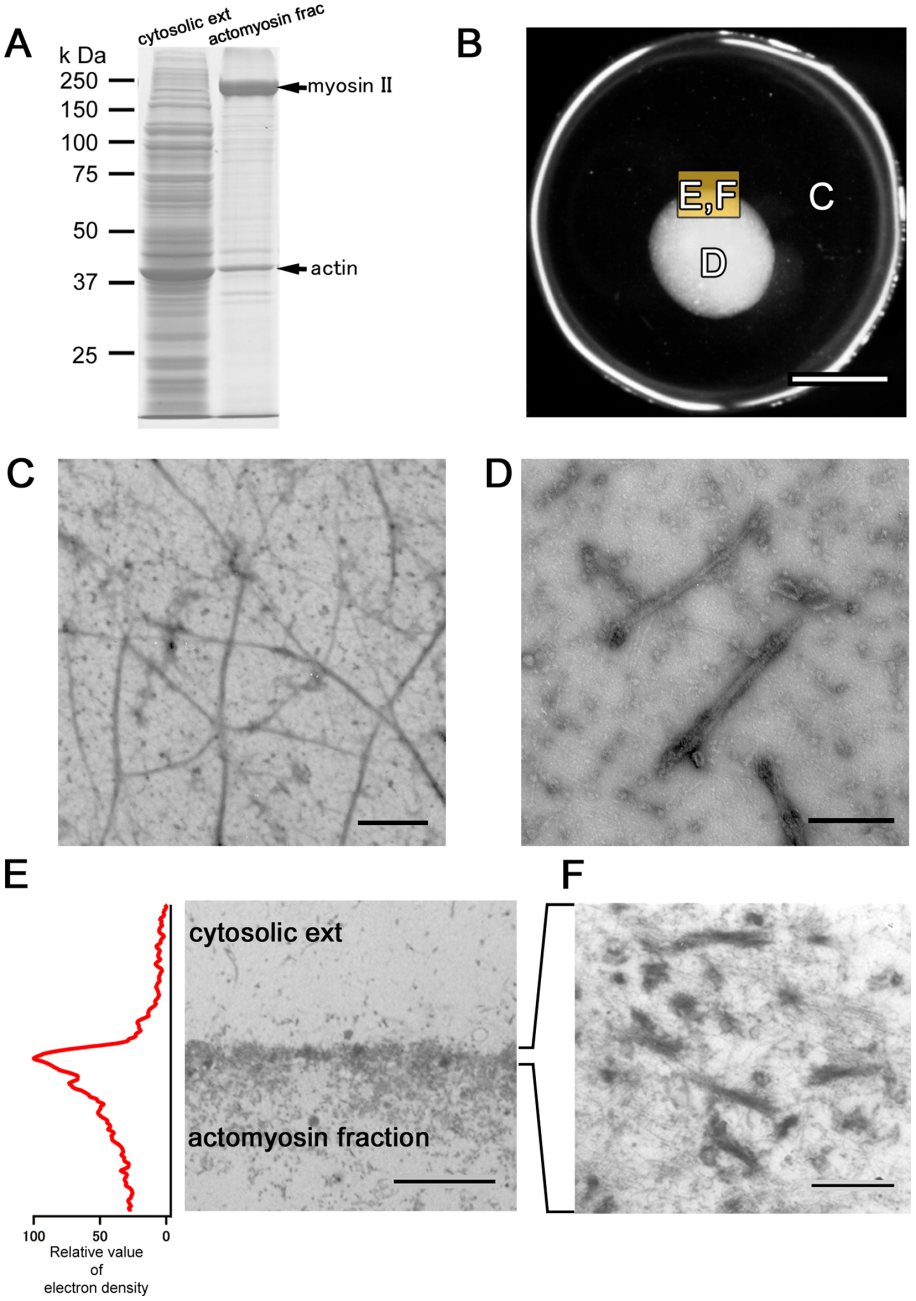
Components of the IVA system. (A) The cytosolic extract contains more actin and little myosin, whereas the actomyosin fraction contains more myosin and moderate actin. Upper arrow indicates myosin II and lower arrow indicates actin. (B) Low-magnification image showing regions in each figure. (C) Electron micrograph showing single and bundle actin filaments in cytosolic extract. Samples were negatively stained with 2% uranyl acetate. Scale bar, 1 µm. (D) Electron micrograph showing actin and myosin filaments in the actomyosin fraction. Scale bar, 200 nm. (E) Electron micrograph showing that the interface between the cytosolic extract and myosin fraction comprises an electron-dense structure. Scale bar, 5 µm. (F) At higher magnification, the boundary surface appears to comprise highly aggregated actin and myosin. Scale bar, 250 nm.

Packed *A. proteus* cells were ultracentrifuged to obtain a cytosolic extract. Because most myosin II is present in thick filament [14], the extract contained little myosin II (Figure 2A and C); however, this protein was abundant in the pellet following high-speed centrifugation. The pellet was extracted with a high-salt solution and the eluate was dialyzed to form myosin II thick filaments. Filaments were collected by centrifugation and suspended in EMP solution to obtain the “actomyosin fraction.” The cytosolic extract was rich in actin and poor in myosin, whereas the actomyosin fraction was rich in myosin with moderate actin (Figure 2A, C and D). Injection of the actomyosin fraction into the cytosolic extract induced vigorous locomotion (Figure 1A, S2, Movie S2 and S3). The boundary between the injected actomyosin fraction and surrounding cytosolic extract bulged to form a sphere (Movie S2 and S3). Spheroidal actomyosin body content effused into the surrounding cytosolic extract from a hole in the boundary, forming a new surface structure on the boundary. Sequential repetition of this contraction and effusion produced locomotion similar to bleb-driven amoeboid locomotion. This model was named the “*in vitro* amoeba” (IVA) system.

Analogous consideration of spherical droplet formation suggests the presence of a membrane-like structure on the boundary and emergence of surface tension. To reveal boundary surface composition, we fixed the preparation just before first effusion of actomyosin body contentand examined it with electron microscopy. The body of the myosin fraction was covered by an electron-dense interface, which appeared to comprise highly aggregated actin and myosin (Figure 2E and F). Time- lapse fluorescent image of actin in IVA revealed that actin accumulated at the boundary surface (Figure 1D and Movie S4). Therefore, the motive force of the IVA system may be generated at the actomyosin cortex. To confirm involvement of actomyosin contraction in IVA system movement, it was treated with actin inhibitors (latrunculin B and cytochalasin D) and a myosin inhibitor (2,3-butanedione monoxime (BDM)); marked motility inhibition was observed (Figure S3). The system did not work when the cytosolic extract was replaced with protein-free buffer (data not shown). Therefore, surface contraction was produced by a dense membrane-like structure of randomly interwoven actomyosin. It is reasonable that the motility of IVA system should depend on the contraction of surface actomyosin, but a possibility that actin dynamics, polymerization and depolymerization, might partly take part in motive force generation cannot be completely excluded. The tension-exerting surface or cortex is similar to that of other actomyosin systems [15], [16]. Bleb locomotion was driven by the contraction force of the actomyosin cortex on the surface. A newly formed pseudopod-like structure passed through four stages (Figure S4): expansion, contraction without elution, resting, and contraction with elution. In the expanding stage, the actomyosin enclosed by the contracting actomyosin boundary surface flowed into a new pseudopod-like structure. In the contraction without elution stage, the effused actomyosin formed a new boundary surface on the cytoplasmic extract and started contracting. With the cortex restricting the actomyosin fraction flux beyond the boundary, coalescence of the cortices along the boundary generated hydrostatic pressure within the actomyosin fraction. In the resting stage, internal hydrostatic pressure was equal to surface contraction-, ambient pressure-, and gravity-generated forces. The round-shaped IVA system shrank to a sphere. During contraction with elution, a weak region of the interface ruptured spontaneously, resulting in effusion of the internal actomyosin fraction into the external cytosolic extract.

The amoeba-derived system reconstituted an amoeboid migration type through pseudopod elongation. Pseudopod-driven locomotion resembles bleb-driven locomotion phenomenologically. Repeated IVA contraction and effusion is similar to oscillatory motion of a bleb in a fibroblast and its fragments [17]. Both dynamics were driven by the contractile nature of the active actomyosin cortex, confirmed by the actin and myosin inhibitor treatments, respectively (Figure S3). IVA is the simplest system for cell blebbing and locomotion [18]. However, the IVA system and previous bleb systems differ in several ways [17], [19]–[21] (larger scale, higher flow speed, and more vigorous motion). The characteristic motions of the IVA system have been briefly described for defining fundamental features of amoeboid-type motion.

**Figure 3:**
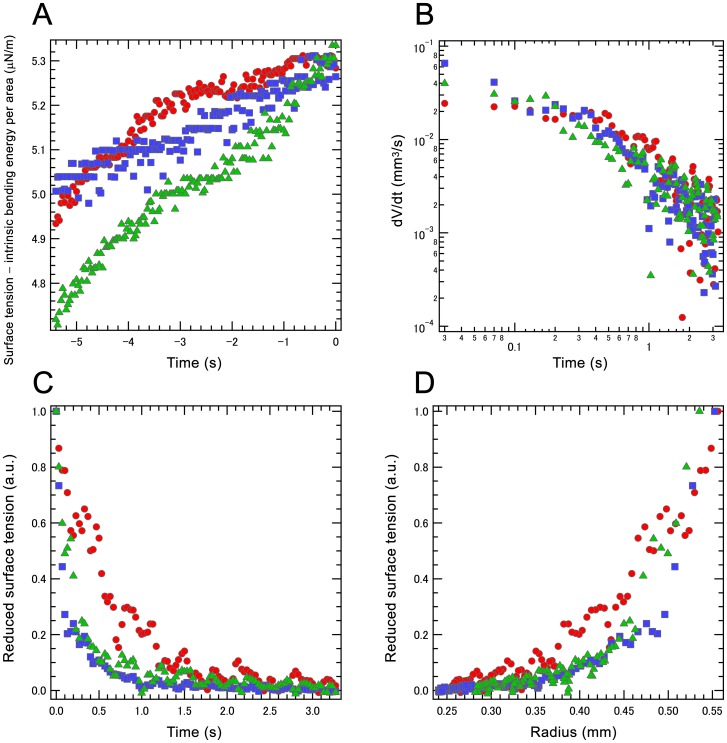
Surface tension in the IVA system. (A) In resting stage, total surface tension on the actomyosin body increases with time. Time 0 indicates initial time of internal actomyosin fraction effusion into external cytosolic extract. (B) Relationship between temporal changes in volume in the contraction with elution stage. (C) Relationship between reduced surface tension and time in the contraction with elution stage. (D) Relationship between reduced surface tension and radius in the contraction with elution stage. Three different typical analyses are shown in all figures.

In expanding and contracting without elution stages, the actomyosin body was transformed from amorphous to a spherical shape, indicating that the generated cortical tension made it spherical. Accordingly, slight shrinking in resting stage reflects transformation from a distorted sphere to sphere. For small distortions, the Mahadevan–Pomeau model can be used [22], which is based on 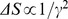, where 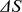 is the deviation of the surface area from that of a sphere and 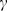 is the total surface tension, including cortical tension and counteracting Young forces from the droplet body and cortex [17], [21], [23]. The model indicates that the total surface tension on the actomyosin body increased in resting stage (Figure 3A) and stage 4 began when the increasing inner pressure exceeded the strength of the actomyosin cortex on the surface [23].

In the contraction with elution stage, if the bulge and original actomyosin body were static, a Laplace-type pressure balance equation could be applied [21], [22]; however, the elution was not static. Accordingly, a dissipation balance was applied for estimating cortical tension development time. The dissipation on the droplet surface can be approximated as 
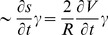
, where 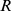, 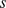 and 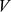 are radius, surface area, and volume of the droplet, respectively. This surface energy is assumed to be used in hydrodynamic dissipation as 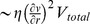 [24], where 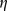 is the viscosity and 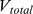 is the total flowing volume, which can be approximated as the conserved total actomyosin volume. Under a linear approximation, the shear strength 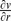 is proportional to the flow speed 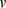. The flow speed may be expressed in this case by that at the effusion hole of 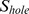 of the magnitude 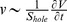. The qualitative balance 
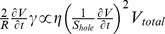
 leads to the simple relation 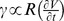. (Eq. 1).

Figure 3B shows the time development of droplet volume measured from the projected droplet area. The experiments exhibit 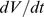 decaying with 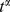, where 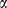 is approximately −1 in this limiting case. Figures 3C and D show the qualitative behavior of total surface tension 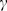 calculated from the experimental data using the equations 1. The surface tension decayed but persisted during shrinking. For the order estimation of the tension, if viscosity 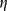 exhibits the same value of water, the shear strength 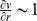 [s^−1^] results in ∼ 20 µN/M in surface tension, which was one order of magnitude larger than that in Figure 3A. From this order estimation and the fact that the actomyosin viscosity was quite high, e.g., 4.5mPa s in cytoplasmic sol *in vivo* [25] and ∼100 mPa s in F-action solution [26], it can be supposed that almost all the contractive force of the actomyosin gel was used for deforming own body [27], and the part of the stored elastic energy was relieved from the rupture as the protrusion. In this geometry, Young’s modulus of the inner droplet part has relatively lower value because of the continuous flow, and Young’s modulus of the cortex 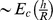, where is the thickness of the cortex, which can be assumed to be lower in the early effusion stage: 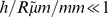 [21]. Although 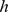 could not be measured, the empty shell exhibited a rather thick cortex with buckling. 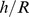 should be considerable in the late contraction stage. Reduced surface tension decreased with constant tendency (Figure 3C); however, the decay exhibited rapid decrease rather than 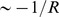. Thus, Figure 3C and D indicate that the actomyosin gel cortex continuously contributed to contraction even after initial relaxation. A static or small bleb may originate in compressive elasticity of the body. The elastic body was compressed to the point of pressure equalization, while the incompressible solution leaked out as a bleb. This balance generated a static or small bleb. However, when the actomyosin body had a nearly sol constitution or non-elastic response in the deformation time scale, the above mentioned balance among surface force, Laplace pressure and Young’s moduli of the inside and the cortex will be broken to take either stationary or effusing [18]. Because some experiments exhibited no effusion, the Young’s modulus could sometimes afford sufficient antagonistic force against cortical tension; however, the cortex thickness and modulus were still uncontrolled. The elastic response or flowability of the inner part plays an important role in generating amoeboid locomotion from blebbing. How IVA acquire adaptive flowability remains to be clarified.

**Figure 4:**
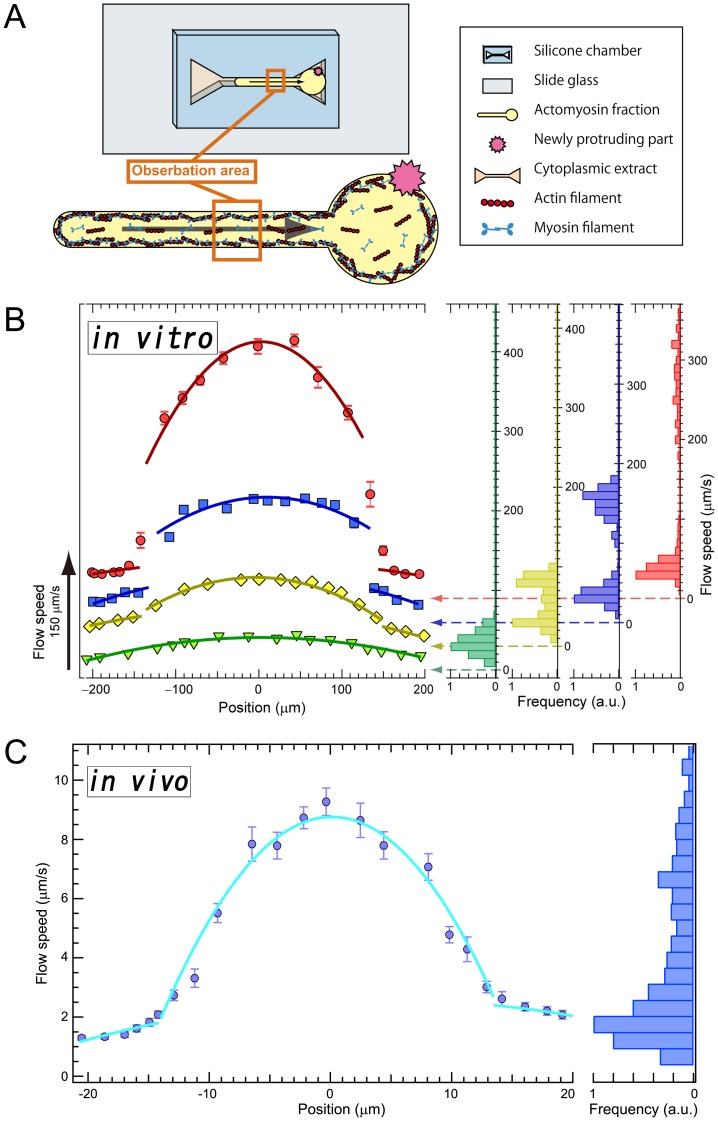
Rheological characterization of the IVA system and living cells. (A) Schematic diagram of *in vitro* analysis. Flow of the actomyosin fraction in the narrow rectangular space was examined using fluorescent microbeads and real-time confocal microscopy. (B) The typical four different velocities of fluorescent bead flow in *in vitro* analysis. In the slowest flow, the scaling exponent was approximately −2 and the histogram shows one peak, whereas the others show two. Error bars show s.e.m. (n = 25 at each point). (C) Flow profile of a living amoeba similar to that of the IVA system in fast flow. Error bars show s.e.m. (n = 20 at each point).

Figure 4 shows a tail retraction motion model exhibiting viscoelastic behavior. A flow chamber of silicone elastomer was immersed in the cytoplasmic extract, and the actomyosin fraction was injected into the chamber (Figure 4A). The actomyosin fraction spread to the thin channel by wetting or capillary attraction and mixed with the cytoplasmic extract to form an actomyosin gel. After formation of a boundary between the actomyosin fraction body and external cytoplasmic extract, the actomyosin body in the narrow channel was filled with the actomyosin gel. The boundary surface form in the trigonal space spontaneously bulged as seen in the IVA system, and the actomyosin fraction in the narrow channel flowed toward the actomyosin body in the trigonal space. The actomyosin gel flow profiles in the narrow channel were examined using fluorescent microbeads for particle image velocimetry with real-time confocal microscopy.

Figure 4B shows four different velocity flows with fluorescent beads. In the slowest flow (maximum speed approximately 41 µm/s, Movie S5, and painting green in Figure 4B and S5), the distribution exhibited the Hagen–Poiseuille flow profile. At higher speeds, the flow was split by the slipping interfaces, also apparent from histograms of the flow speed showing two peaks for each faster flow (Movie S6–S8, Figure 4B and S5). In the split flow profiles, inner and outer flows can be fitted by 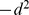 lines (*d* is displacement from the center). Because myosin thick filaments possess many bipolar actin-binding sites, actin and myosin filaments continually bind and release each other in the presence of ATP [27]. This release allows recombination of the gel network under low shear so that in the presence of ATP, the actomyosin gel may exhibit fluidic behavior under a low shear rate despite being a gel phase. In contrast, under fast flow or high shear rate, the connections between actin and myosin are broken by shear stress. Once the network broke, a percolation network of the actomyosin gel did not emerge during the motion (Figure S6). This slipping plane breaking or formation began at approximately 0.5 s^−1^ and was maintained in regions with higher rates (2–4 s^−1^). Figure 4C and movie S9 show *in vivo* measurement in living amoeba using cell staining by MitoTracker; a slipping plane formed above approximately 0.25 s^−1^ and the shear strain around the slipping plane was approximately 2 s^−1^.

In the recent study, it is reported that a fibrous actin solution exhibits shear thinning or shear banding property [26]. Although the mechanism of that behavior on actin solution has not been solved, the similar discussion may be applicable to the present results, i.e., the bipolar myosin thick filament and actin filaments should be forced in parallel to the stream line to reduce friction or entanglement each other, which may inhibit the growth of gelling network. At least, maintenance of the strongly sheared region in the IVA might be explained by the above mechanism, where they have an interesting result in common that the yielded shear stress of the actin fiber solution was the same order of magnitude as that in the present IVA gel and also *in vivo* amoeba. However the first stage in the flow response in IVA should be discussed from an aspect of active gel as in above mentioned paragraph because the F-actin solution exhibit shear bands stating from 0.1 s^−1^ or the lower shear rate, which is qualitatively different from the present results. Toyota *et al*. have measured the active actomyosin gel by use of the micro-rheology method, that the active gel exhibits characteristic viscoelasticity with faster diffusion in the low-frequency region (in slower time scale) and with normal viscoelasticity in the high-frequency region [27]. The intermediate time scale was indicated around the order of 1 to 10 [s] accompanied with the suggestion of the myosin running speed ∼10 nm/s and its progressive length ∼100 nm. The present threshold value of 0.5 [s^−1^] in shear rate shows interesting correspondence to the scale of the spontaneous motion of the bridging points, i.e., 100 nm with 10 nm/s speed. As a hypothesis, slower shear or transformation could be followed by the active-gels through the detachment and re-attachment of the gel-bridging points with myosin motors. In other words, slower transformation may not be serious for the active-gel compared to its own stress or microscopic transformation. However, it is inferred that active-gels exhibit elastic responses against the faster shear or transformation, which results in breaking of the gel to separate the regions with different flow speeds. These properties would be useful in an amoeboid locomotion as discussed as follows.

Cytoplasmic sol–gel conversion is important in many cellular processes, e.g., the elastic response of the gel-like body of the cell is essential to stop bleb growth [18]. When the elastic structure inside a cell cannot withstand pressure from the active cortex, the entire cytoplasm is spontaneously carried into a bleb (Figure S2 and Movie S2). A cell whose entire cytoplasm enters a rigid gel state could not migrate with amoeboid locomotion. Accordingly, cytoplasmic sol and gel distribution must be regulated by intra- and extracellular environments. The intrinsic nature of the sol–gel transition induced by external stress, as shown in IVA, would be useful in regulating cell migration in a primitive manner. Using this, an amoeba can avoid complex regulation by relying on autonomous stress- or flow-regulated transition. Although the importance of strain localization or dynamic sol–gel conversion of the cytoplasm in the regulation of cellular processes has not been recognized, several studies support cytoplasmic shear thinning in amoebae [25], [28] and shear banding of F-actin solution [26]. Intracellular calcium ion concentrations regulate cytoplasmic sol–gel conversion [7] in which actin-binding proteins and calcium ions control sol–gel states, with a high concentration of calcium ion generally inducing a sol state and a low concentration inducing a gel state. Accordingly, calcium ion concentrations should be high in the sol region and low in the gel region of the cell. However, reports on calcium ion distribution in cells are conflicting. High calcium ion concentrations in posterior regions [29]–[31], in anterior regions [1], [31], and low concentrations throughout the cell [32] have been reported. This can be partly explained by our hypothesis of mechanically responsive sol–gel transition, at least in amoeboid migration. If the cytoplasm has intrinsic nature of mechanically responsive sol–gel conversion, it can behave as an elastic gel under static conditions. However, rupture of the static balance causes dynamic protrusive excursion, as observed in our model system, through a mechanical gel-to-sol transition. In this motion, signals containing calcium ion would constitute triggers or controls on the cortex, affecting strength, thickness, or activity to break symmetry for determining the migrating direction.

Thus, we have provided new insights into cytosolic sol–gel conversion mechanisms, in which an active actomyosin cortex system exhibits mechanically responsive viscoelasticity. This physical property is sufficient to explain cytosolic sol–gel conversion during amoeboid motion. Although almost all our results were observed in *in vitro* systems, they suggest that organisms use signaling regulation and this unique physical property of the active cortex.

## Supporting Information

Figure S1 **Cytoplasmic sol–gel distribution** ***in vivo*** **.** (A) Normal locomotion of amoeba. Trajectories of cytoplasmic particle flow are shown in (B) over 1 s, and (C) is a merged image of (A) and (B). A scheme for the distribution of cytoplasmic sol and gel is shown in (D). Amoeba cytoplasm has two layers of sol and gel, in which the sol layer is enclosed by the gel layer. Scale bar, 50 µm.
(TIF)

Figure S2 **Locomotion in the** ***in vitro*** **amoeba (IVA) system.** When an actomyosin fraction is injected into the cytosolic extract, the actomyosin fraction displays movement similar to bleb-driven amoeboid locomotion. Temporal changes in movements of pseudopod-like structures are shown in the trace. Scale bar, 1 mm.
(TIF)

Figure S3 **Inhibition of the** ***in vitro*** **amoeba (IVA) system movement.** IVA system movement was stopped by treating with actin and myosin inhibitors. Arrows indicate the time of formation of new pseudopod-like structures. The experiments were repeated at least six times, yielding essentially identical results. One typical example of individual inhibitors is shown in the figure.
(TIF)

Figure S4 **Four stages in pseudopod-like structure activity.** Middle graph shows the area transition of pseudopod-like structure, which is represented as green in the upper real image and yellow–green in the lower schematic diagram. The pseudopod-like structure shows four stages of activity: expanding, contracting without elution, resting, and contracting with elution. In the expanding stage, the actomyosin enclosed by the contracting actomyosin boundary surface flows into a new pseudopod-like structure, increasing its volume. In the stage of contracting without effusion, the newly effused actomyosin forms a new boundary surface on the cytoplasmic extract and begins to contract, restricting water flux as the increased pressure generates a dense, actomyosin-rich interface and leading to increased hydrostatic pressure within the actomyosin fraction. In the resting stage, the internal hydrostatic pressure is equal to the force produced by boundary surface contraction so that the IVA system appears not to move. In the stage of contracting with elution, weak regions of the interface fail, resulting in effusion of the internal actomyosin fraction into the external actin-rich cytosolic extract.
(TIF)

Figure S5 **The trajectory pattern in different flow rates.** The actomyosin fraction flow profiles in the narrow channel were examined using fluorescent microbeads. Different colors represent difference of flow speed corresponding to the color in Figure 4B. In the slowest flow (green), the flow exhibited the Hagen–Poiseuille type flow. At higher speeds (yellow, blue and red), the flow are split by the slipping interfaces.
(TIF)

Figure S6 **A new mechanism of cytosolic sol–gel conversion.** With actin and myosin filaments repeatedly binding and releasing each other in the presence of ATP, an actomyosin solution behaves as an active gel in which the actomyosin gel may behave as a fluid under a small shear rate despite being a gel phase. In contrast, in fast flow or under a large shear rate, the connections between actin and myosin are broken by shear stress, producing a slipping plane.
(TIF)

Movie S1 **Motility of a normal locomoting** ***Amoeba proteus*** **.** Amoeba cytoplasm has two layers of sol and gel, in which the sol layer is enclosed by the gel layer. During locomotion, the sol layer of the cytoplasm flows in the direction of locomotion, whereas the gel layer is fixed. As the cytoplasm stops moving, the sol in the anterior region is converted to gel. This movie is in normal speed. Scale bar, 50 µm.
(MP4)

Movie S2 **Motility in the IVA system.** When the actomyosin fraction is injected into the cytosolic extract, the actomyosin fraction displays movement similar to bleb-driven amoeboid locomotion. Initially, the boundary between the injected actomyosin fraction and surrounded cytosolic extract bulges into a sphere. The contents of the spheroidal actomyosin body then effuse into the surrounding cytosolic extract from the hole in the boundary. The effused actomyosin fraction forms a new surface structure on the boundary. Sequential repetition of these contraction and effusion events leads to locomotion similar to bleb-driven amoeboid movement. This movie is in normal speed. Scale bar, 1 mm.
(MP4)

Movie S3 **Motility in the IVA system.** An experiment different from Movie S2. Because the activity of blebbing in IVA is different in different preparation of experiment and similar in the same preparation, the differences of activity is probably due to the activity of living cells. Since the period of elution and contraction cycle is between ∼1 s to 30 s in IVA system. This movie is in normal speed. Scale bar, 1 mm.
(MP4)

Movie S4 **Time-laps fluorescence image of actin in the IVA system.** Just after injecting actomyosin fraction into cytoplasmic extract, actin was spread in entire IVA system. After a few tens of seconds, the spread actin filament contracted each other to shrink the body. The accumulated actin showed dense surface and inhomogeneous inner part, which might reflect the gel density. At the time of forming a new pseudopod like structure, a part of the boundary surface actin was broken (arrow head) and actomyosin body content effused into the surrounding cytosolic extract from the broken boundary surface. This movie is in 5×normal speed. Scale bar, 500 µm.
(MP4)

Movie S5 **The flow of the IVA system in chamber (Maximum speed 41 µm/s).** In this speed, flow exhibited Hagen-Poiseuille flow Profile. This movie is in normal speed. Scale bar, 50 µm.
(MP4)

Movie S6 **The flow of the IVA system in chamber (Maximum speed 82 µm/s).** In this speed, the flow was started to split by the slipping interfaces. This movie is in normal speed. Scale bar, 50 µm.
(MP4)

Movie S7 **The flow of the IVA system in chamber (Maximum speed 155 µm/s).** In this speed, the flow was split by the slipping interfaces. This movie is in normal speed. Scale bar, 50 µm.
(MP4)

Movie S8 **The flow of the IVA system in chamber (Maximum speed 304 µm/s).** In this speed, the flow was split by the slipping interfaces. This movie is in normal speed. Scale bar, 50 µm.
(MP4)

Movie S9 **The cytoplasmic flow in living amoeba.** Intercellular flow of living amoeba was visualized by MitoTracker. This movie is in normal speed. Scale bar, 10 µm.
(MP4)
