# Supplementary figures and images for: Reconstruction of Active Regular Motion in Amoeba Extract: Dynamic Cooperation between Sol and Gel States
Source: PLoS One. 2013 Aug 5;8(8):e70317. doi: 10.1371/journal.pone.0070317 (PMC3734023; doi:10.1371/journal.pone.0070317)

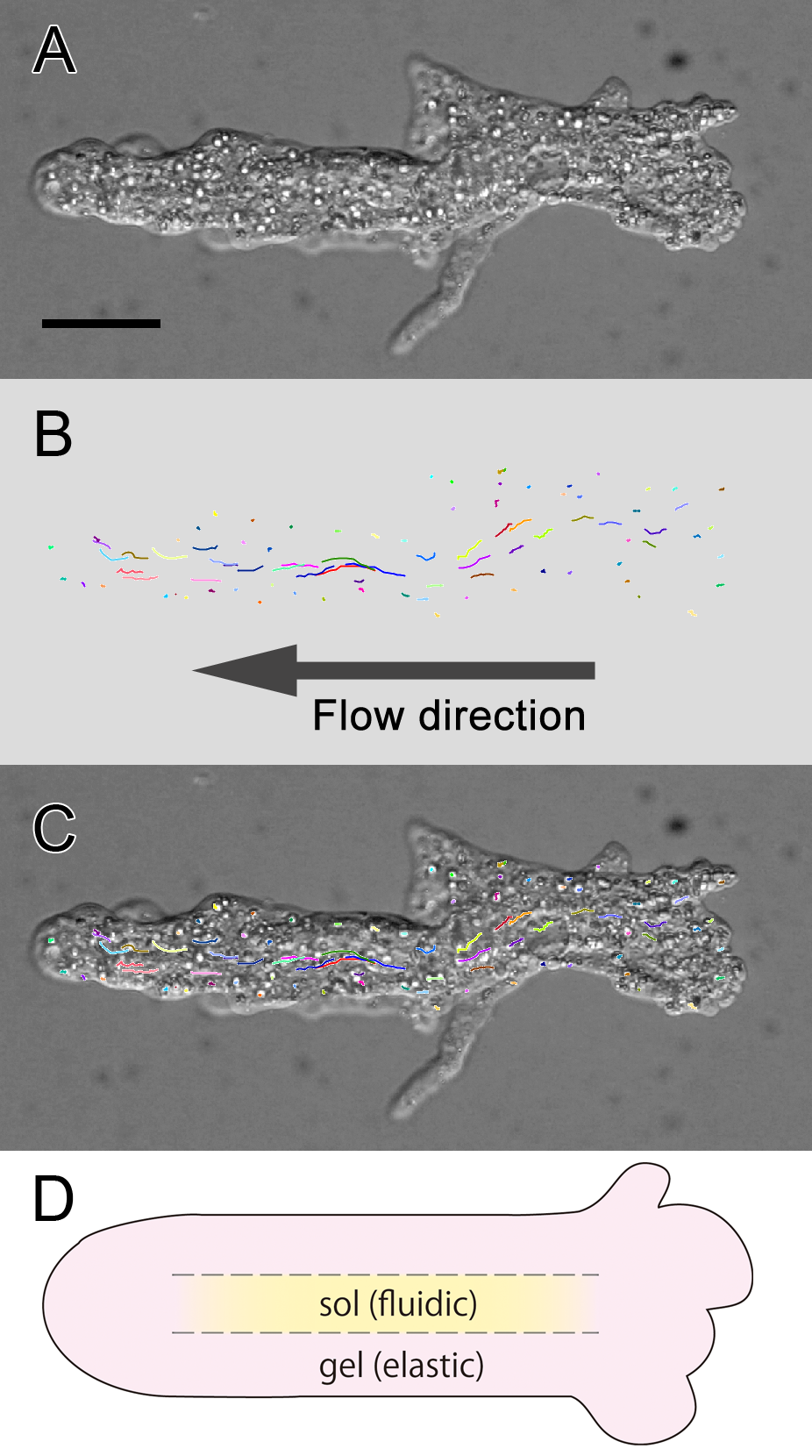

Supplement: Figure S1 — Cytoplasmic sol–gel distribution in vivo . (A) Normal locomotion of amoeba. Trajectories of cytoplasmic particle flow are shown in (B) over 1 s, and (C) is a merged image of (A) and (B). A scheme for the distribution of cytoplasmic sol and gel is shown in (D). Amoeba cytoplasm has two layers of sol and gel, in which the sol layer is enclosed by the gel layer. Scale bar, 50 µm. (TIF) [file pone.0070317.s001.tif]

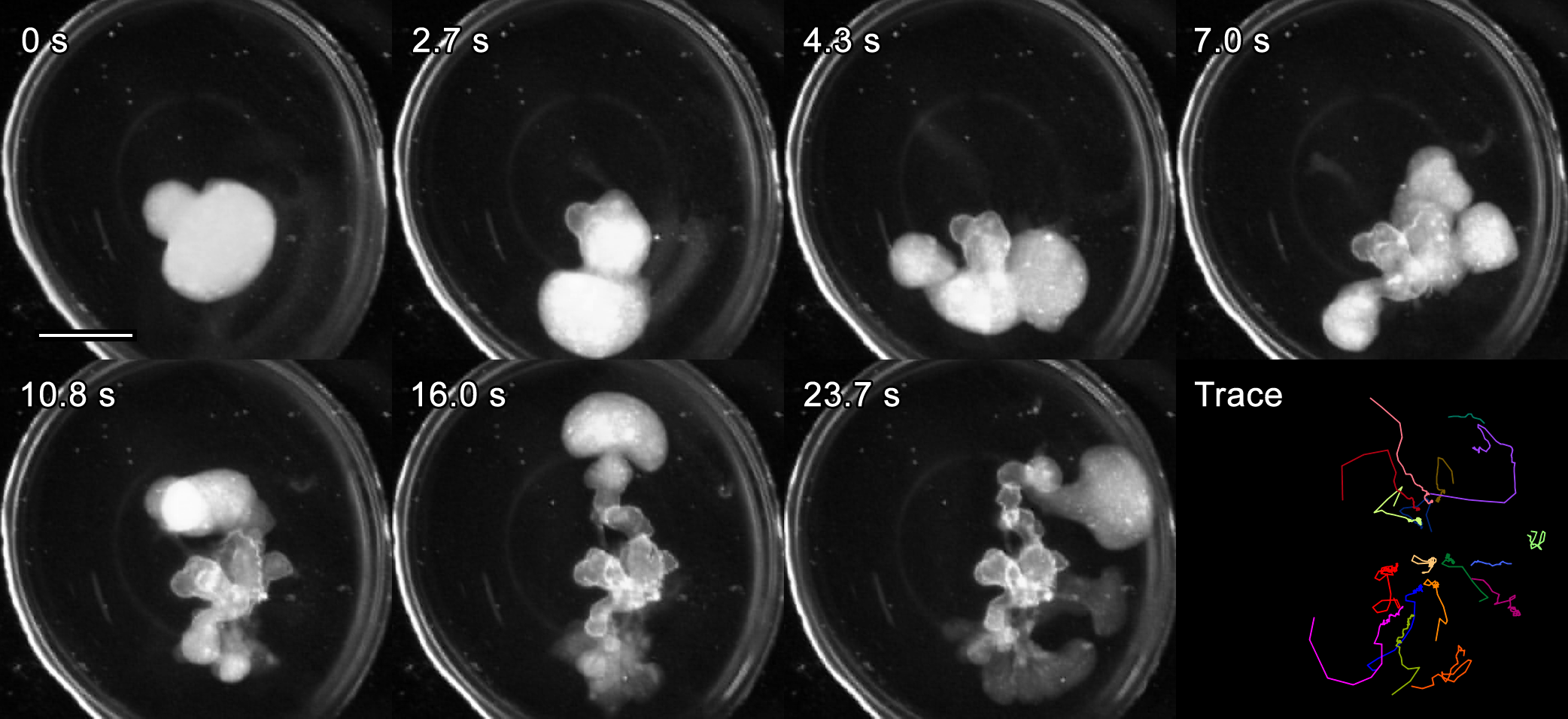

Supplement: Figure S2 — Locomotion in the in vitro amoeba (IVA) system. When an actomyosin fraction is injected into the cytosolic extract, the actomyosin fraction displays movement similar to bleb-driven amoeboid locomotion. Temporal changes in movements of pseudopod-like structures are shown in the trace. Scale bar, 1 mm. (TIF) [file pone.0070317.s002.tif]

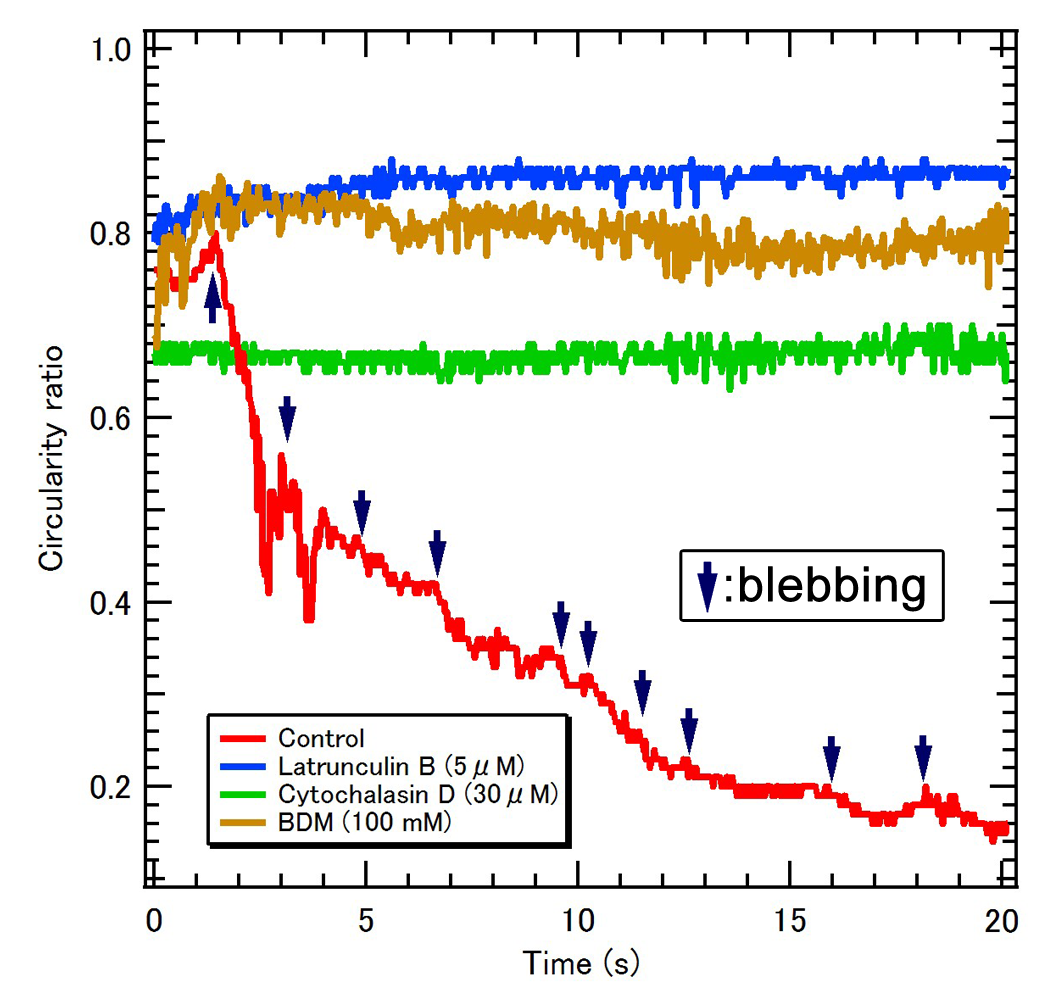

Supplement: Figure S3 — Inhibition of the in vitro amoeba (IVA) system movement. IVA system movement was stopped by treating with actin and myosin inhibitors. Arrows indicate the time of formation of new pseudopod-like structures. The experiments were repeated at least six times, yielding essentially identical results. One typical example of individual inhibitors is shown in the figure. (TIF) [file pone.0070317.s003.tif]

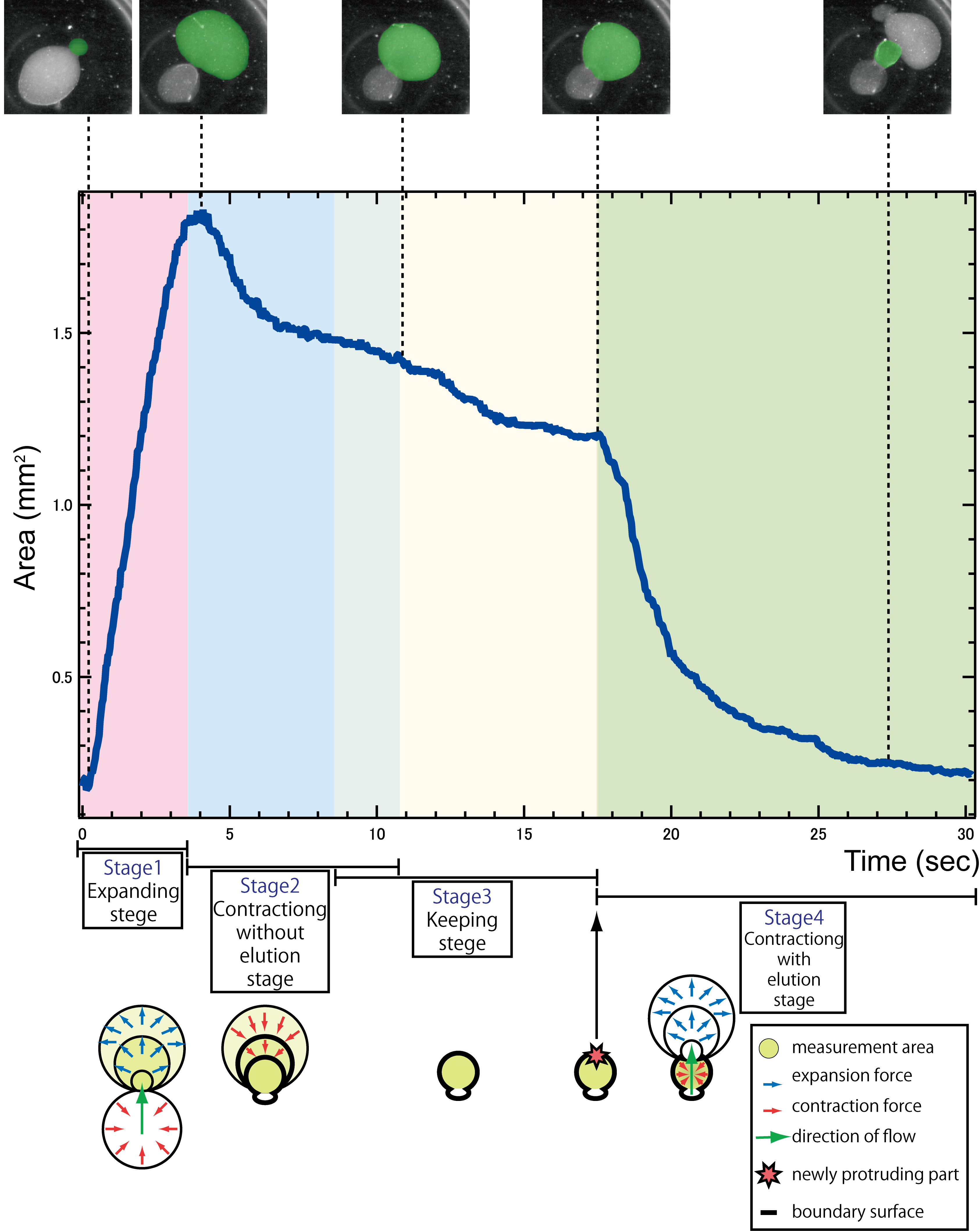

Supplement: Figure S4 — Four stages in pseudopod-like structure activity. Middle graph shows the area transition of pseudopod-like structure, which is represented as green in the upper real image and yellow–green in the lower schematic diagram. The pseudopod-like structure shows four stages of activity: expanding, contracting without elution, resting, and contracting with elution. In the expanding stage, the actomyosin enclosed by the contracting actomyosin boundary surface flows into a new pseudopod-like structure, increasing its volume. In the stage of contracting without effusion, the newly effused actomyosin forms a new boundary surface on the cytoplasmic extract and begins to contract, restricting water flux as the increased pressure generates a dense, actomyosin-rich interface and leading to increased hydrostatic pressure within the actomyosin fraction. In the resting stage, the internal hydrostatic pressure is equal to the force produced by boundary surface contraction so that the IVA system appears not to move. In the stage of contracting with elution, weak regions of the interface fail, resulting in effusion of the internal actomyosin fraction into the external actin-rich cytosolic extract. (TIF) [file pone.0070317.s004.tif]

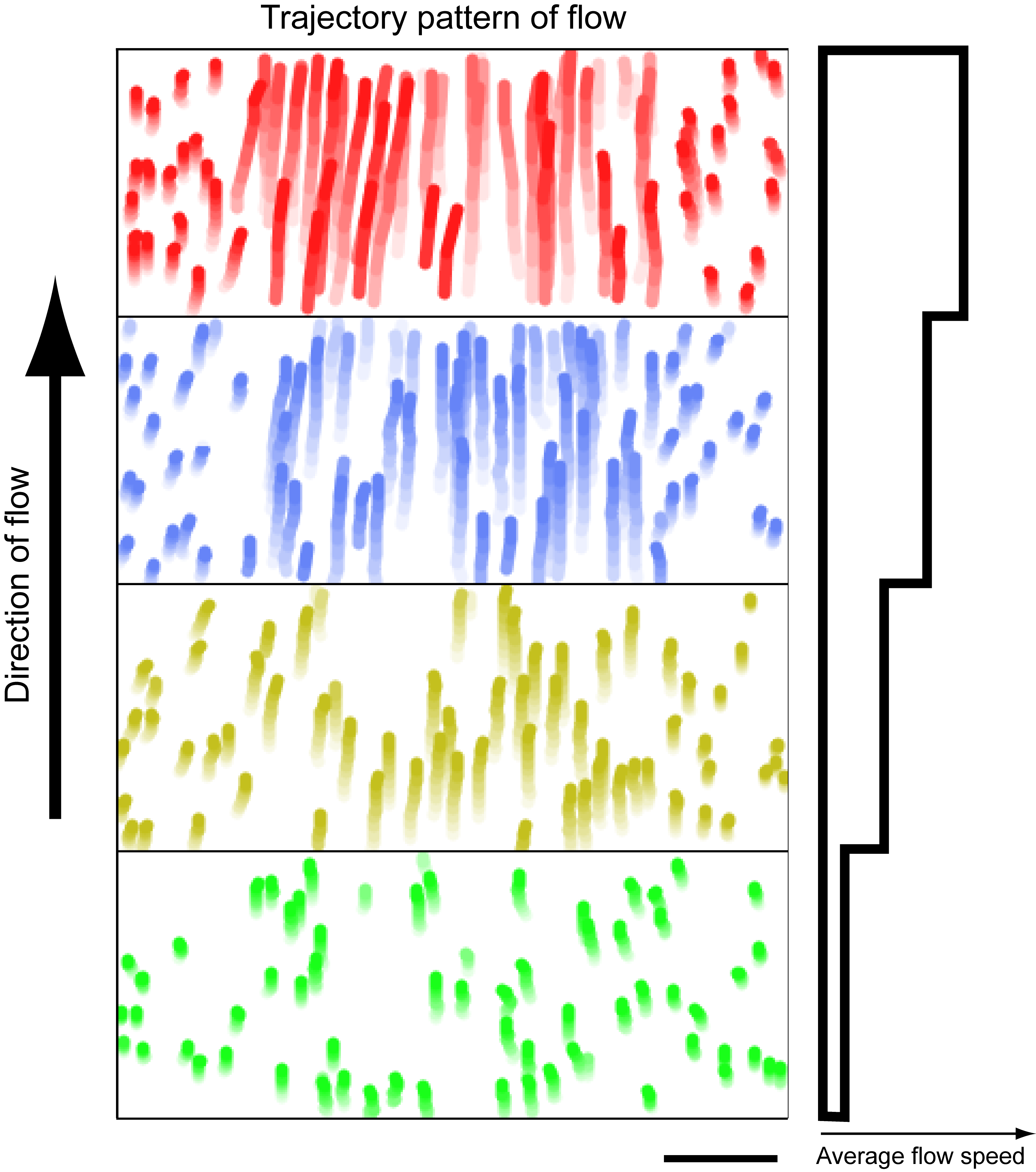

Supplement: Figure S5 — The trajectory pattern in different flow rates. The actomyosin fraction flow profiles in the narrow channel were examined using fluorescent microbeads. Different colors represent difference of flow speed corresponding to the color in Figure 4B. In the slowest flow (green), the flow exhibited the Hagen–Poiseuille type flow. At higher speeds (yellow, blue and red), the flow are split by the slipping interfaces. (TIF) [file pone.0070317.s005.tif]

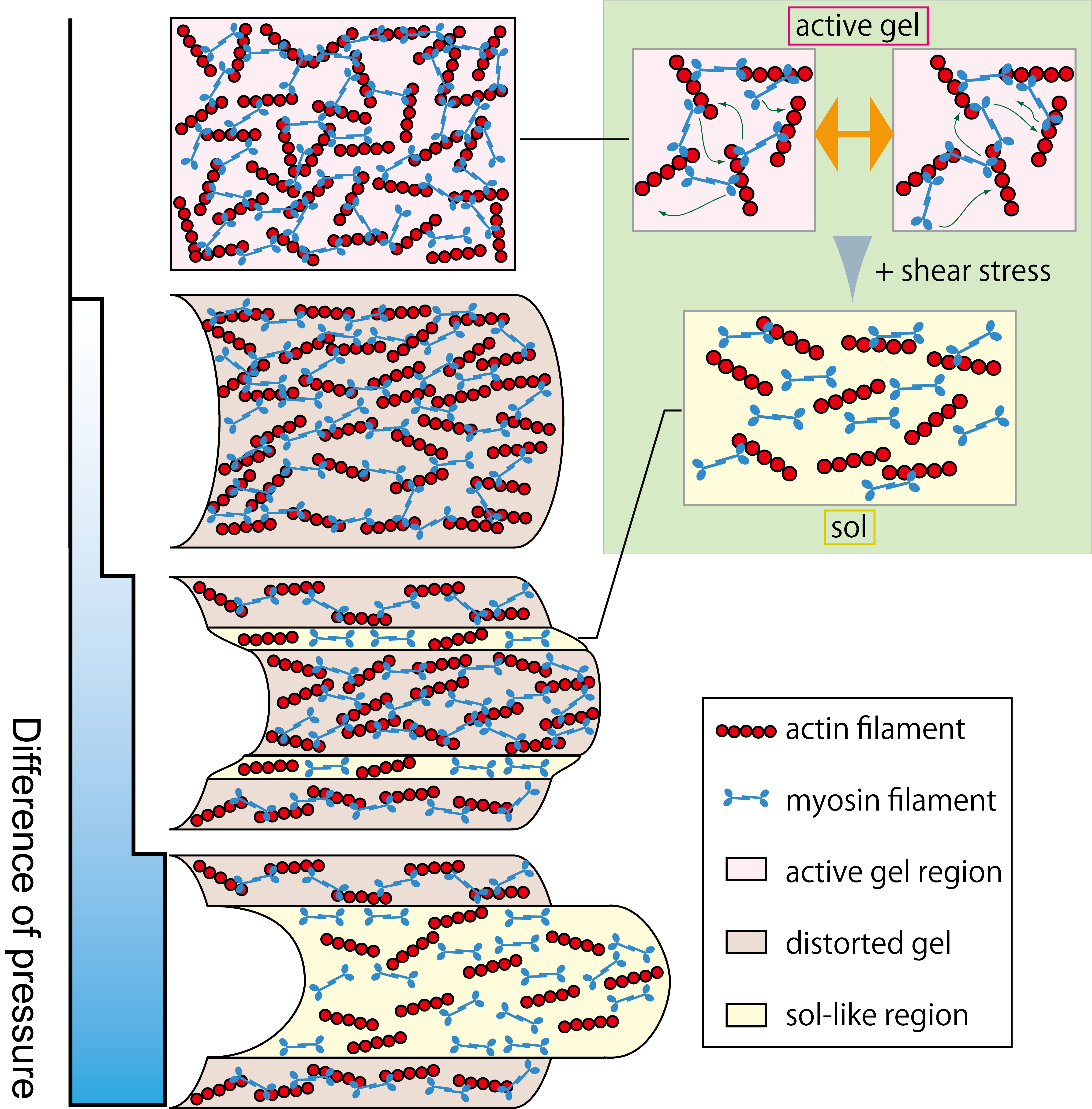

Supplement: Figure S6 — A new mechanism of cytosolic sol–gel conversion. With actin and myosin filaments repeatedly binding and releasing each other in the presence of ATP, an actomyosin solution behaves as an active gel in which the actomyosin gel may behave as a fluid under a small shear rate despite being a gel phase. In contrast, in fast flow or under a large shear rate, the connections between actin and myosin are broken by shear stress, producing a slipping plane. (TIF) [file pone.0070317.s006.tif]
